# Blocking Fibroblast Growth Factor Receptor Signaling Inhibits Tumor Growth, Lymphangiogenesis, and Metastasis

**DOI:** 10.1371/journal.pone.0039540

**Published:** 2012-06-25

**Authors:** Frédéric Larrieu-Lahargue, Alana L. Welm, Marion Bouchecareilh, Kari Alitalo, Dean Y. Li, Andreas Bikfalvi, Patrick Auguste

**Affiliations:** 1 Laboratoire de l’Angiogenèse et du Microenvironement des Cancers, Université de Bordeaux, Talence, France; 2 INSERM U 1029, Talence, France; 3 Program in Molecular Medicine, University of Utah, Salt Lake City, Utah, United States of America; 4 Department of Oncological Sciences, Huntsman Cancer Institute, University of Utah, Salt Lake City, Utah, United States of America; 5 Molecular Cancer Biology Program, Biomedicum Helsinki, Department of Pathology, Haartman Institute and Helsinki University Central Hospital, University of Helsinki, Helsinki, Finland; King Faisal Specialist Hospital & Research center, Saudi Arabia

## Abstract

Fibroblast Growth Factor receptor (FGFR) activity plays crucial roles in tumor growth and patient survival. However, FGF (Fibroblast Growth Factor) signaling as a target for cancer therapy has been under-investigated compared to other receptor tyrosine kinases. Here, we studied the effect of FGFR signaling inhibition on tumor growth, metastasis and lymphangiogenesis by expressing a dominant negative FGFR (FGFR-2DN) in an orthotopic mouse mammary 66c14 carcinoma model. We show that FGFR-2DN-expressing 66c14 cells proliferate *in vitro* slower than controls. 66c14 tumor outgrowth and lung metastatic foci are reduced in mice implanted with FGFR-2DN-expressing cells, which also exhibited better overall survival. We found 66c14 cells in the lumen of tumor lymphatic vessels and in lymph nodes. FGFR-2DN-expressing tumors exhibited a decrease in VEGFR-3 (Vascular Endothelial Growth Factor Receptor-3) or podoplanin-positive lymphatic vessels, an increase in isolated intratumoral lymphatic endothelial cells and a reduction in VEGF-C (Vascular Endothelial Growth Factor-C) mRNA expression. FGFs may act in an autocrine manner as the inhibition of FGFR signaling in tumor cells suppresses VEGF-C expression in a COX-2 (cyclooxygenase-2) or HIF1-α (hypoxia-inducible factor-1 α) independent manner. FGFs may also act in a paracrine manner on tumor lymphatics by inducing expression of pro-lymphangiogenic molecules such as VEGFR-3, integrin α9, prox1 and netrin-1. Finally, *in vitro* lymphangiogenesis is impeded in the presence of FGFR-2DN 66c14 cells. These data confirm that both FGF and VEGF signaling are necessary for the maintenance of vascular morphogenesis and provide evidence that targeting FGFR signaling may be an interesting approach to inhibit tumor lymphangiogenesis and metastatic spread.

## Introduction

Fibroblast Growth Factors (FGFs), which signal through FGF receptors (FGFR-1-5), are involved in a broad range of biological processes such as migration, tubulogenesis, proliferation, and differentiation of various cell types [1]. Evidence shows that FGF signaling promotes tumor development and metastasis by directly regulating cancer cell proliferation, survival and tumor angiogenesis [2], [3], [4], [5].

The lymphatic system is a blind-ended network of endothelial cell-lined vessels that maintains fluid homeostasis by unidirectionally transporting tissue fluid, extravasated plasma proteins, lipids and cells from the interstitial space to the circulatory system via the thoracic duct. Several studies have demonstrated the importance of the lymphatic system as a route for tumor dissemination [6] and that metastasis is enhanced by VEGF-C via an increase in tumor lymphangiogenesis [7], [8], [9]. FGF-2 has also been shown to indirectly induce lymphangiogenesis, *in vivo*, in a mouse cornea assay by upregulating VEGF-C [10]. FGF2 also has been shown *in vitro* to act directly on lymphatic endothelial cell migration, proliferation and tubulogenesis [11], [12].

However, no study has ever addressed the role of FGFR signaling in tumor lymphangiogenesis and metastasis via the lymphatic system. Here, we provide evidence that blockade of FGFR signaling in tumor cells using dominant negative FGFR (FGFR-2DN) approach [4], [13], [14], impairs mammary carcinoma growth and metastasis, leading to an improvement in overall survival. Blockade of FGFR signaling causes a decrease in tumor lymphangiogenesis, an increase in isolated lymphatic endothelial cell number and a reduction of VEGF-C expression in tumor cells. Decreased production of VEGF-C is independent of downregulation of other known regulators; COX-2, HIF-1α and PDGF-B [15], [16], [17]. Furthermore, we demonstrate that FGF signaling might also act directly on the tumor lymphatic endothelium by inducing the expression of lymphangiogenesis-related genes. Our results demonstrate that FGFR signaling, in addition to mediating tumor growth, regulates tumor metastasis and lymphangiogenesis via a VEGF-C-dependent mechanism.

**Figure 1:**
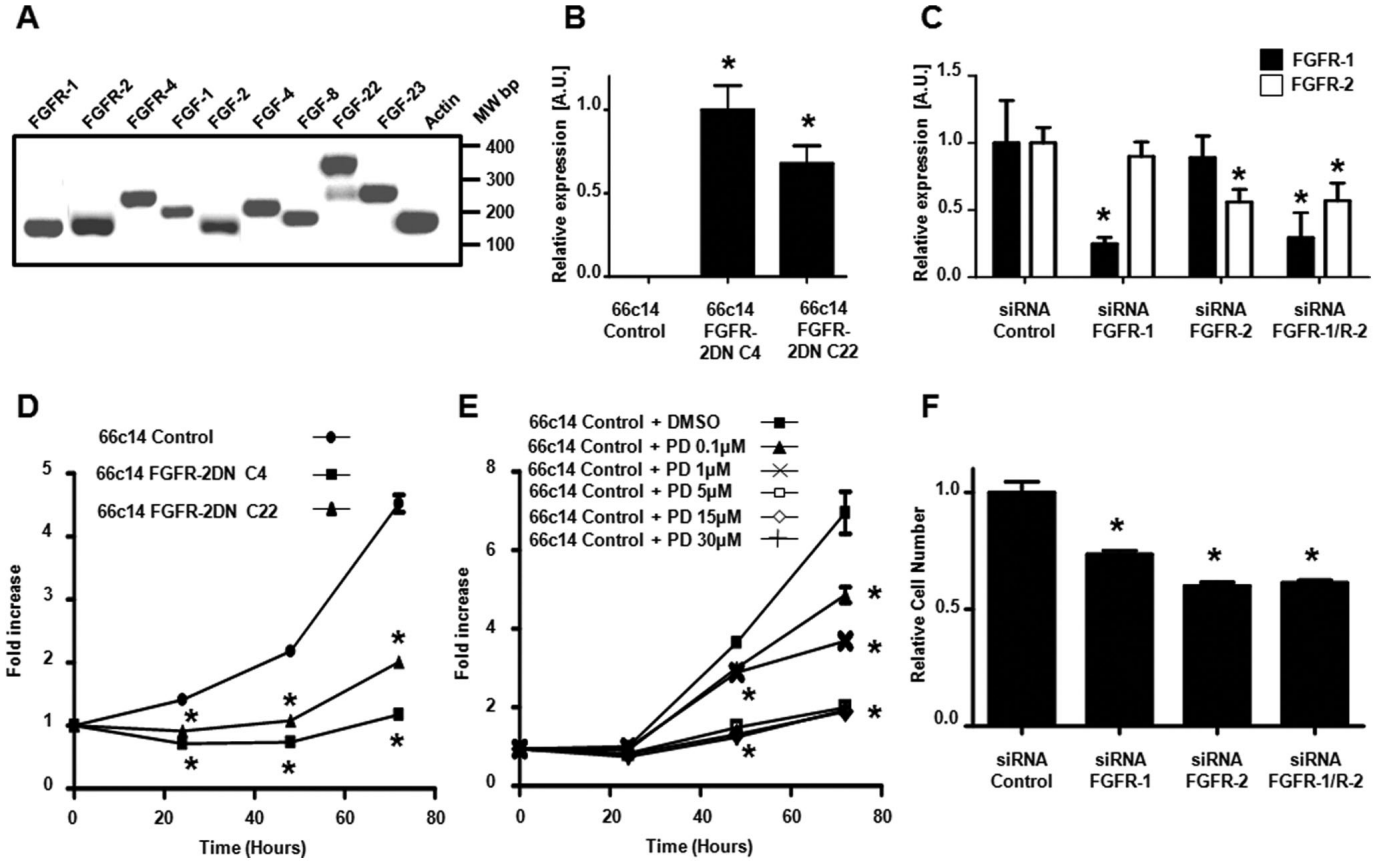
Inhibition of Fibroblast Growth Factor activity by both a genetic and pharmacological approach blocks mouse mammary 66c14 carcinoma tumor cell proliferation. (A) Expression pattern of FGF ligands and receptors mRNA in 66c14 carcinoma tumor cells was determined by standard RT-PCR. (B) Expression of the FGFR-2DN mRNA, receptor truncated for its intracellular tyrosine kinase domain, is only detected in mouse mammary 66c14 carcinoma cells stably transfected with the FGFR-2DN construct (clones C4 and C22) but not with the empty plasmid (Control). (C) Expression of FGFR-1 (black) and FGFR-2 mRNA (white) was determined by quantitative RT-PCR in 66c14 carcinoma cells transfected with control, FGFR-1, FGFR-2 or both FGFR-1 and FGFR-2 (FGFR-1/R-2) siRNA. (D) *In vitro* cell proliferation is inhibited in FGFR-2DN-expressing 66c14 cells compared to mock-transfected cells (Control). (E) 66c14 (Control) carcinoma cells proliferation *in vitro* is decreased when treated with increasing doses of the FGFR inhibitor, PD-173074. (F) Cell proliferation is decreased in FGFR-1, FGFR-2 and both FGFR-1/R-2 siRNA-transfected 66c14 carcinoma cells as compared to control siRNA condition. (*p<0.05 versus respective control group).

## Materials and Methods

### Cell Culture and Reagents

Mouse 66c14 mammary carcinoma and rat C6 glioma cancer cells were provided by Dr Gary Sahagian (Tufts University, USA) and Paul Canioni (University Bordeaux 2, France) respectively. Stable cell clones constitutively expressing a mouse FGFR-2 truncated for its intracellular Tyrosine Kinase domain, and acting as a dominant negative receptor (also called FGFR-2DN), were obtained and cultured as previously described [4], [5]. The three isolated FGFR-2DN-expressing clones were named “C4, C18 and C22” and “3B8, 2A7 and C18” for 66c14 and C6 cancer cells respectively. Empty plasmid-transfected “66c14 control C1–C3” or “BH2” cells were used as expression controls for 66c14or C6 conditions respectively. Human dermal lymphatic microvascular endothelial cells (HMVEC-dLys) were obtained from Lonza and cultured according to manufacturer’s instructions.

FGF-2, VEGF-A, VEGF-C, VEGFR-2/Fc and VEGFR-3/Fc recombinant proteins are from R&D Systems and COX-2 (NS-398), FGFR (PD-173074), HIF-1α (400083) inhibitors are respectively from Cayman Chemical, Calbiochem and EMD Biosciences.

Cobalt chloride (Sigma Aldrich, c8661) was a gift from Dr Sandra Sena, and was dissolved directly in treatment media and sterile-filtered before use.

### Animals and *in vivo* experiments

*In vivo* tumor growth and metastasis experiments were performed as previously described [4], [18], [19].

#### Orthotopic transplantation of mouse mammary carcinoma cells

200,000 66c14 cells, containing a pool of three clones per condition (clones C1–3 or C4, C18 and C22 for control and FGFR-2DN group respectively) or parental cells were injected directly into the exposed inguinal mammary fat pad of anesthetized 6–8 week old female Balb/C mice (The Jackson Laboratory). Tumor volume was measured once a week using a caliper and calculated according to the formula V =  ([major axis] x [minor axis]^2^×(π/6)). Five weeks post injection (tumor size less than 2000 mm^3^), mice were euthanized in a CO_2_ chamber, lungs inflated with 4% formalin and the number of metastatic nodules then quantified.

#### Xenografting of C6 glioblastoma cancer cells

Rat C6 glioma cells were injected subcutaneously into the midline of the back of 8–10 week old-immunodeficient RAG 2/γc mice (Gift of Dr J. P. Di Santo, Institut Pasteur, Paris) as previously described [4].

To study the role of FGFs in *in vivo* lymphangiogenesis, 66c14 tumors were harvested at the time no statistical difference of tumor size was detected, to rule out any tumor growth variation-related vascular changes, and cut in two equal pieces. The first tumor half underwent immunohistology processing (OCT-Tissue-Tek embedded) while the second half was snap frozen for RNA extraction. Half-cut C6 tumors were paraffin embedded, while the second part was snap frozen for RNA preparation.

Survival time is indicated by time to the ethical endpoint, at which time the animals were humanely euthanized.

All studies were repeated twice to ensure reproducibility (with a minimum of 6 mice per group).

Animal experiments were conducted in accordance with the University of Bordeaux and University of Utah Institutional Animal Care and Use Committees.

### *In vitro* Assays

*In vitro* proliferation assays were performed as previously described with minor modifications [19].

Parental 66c14 cells were sequentially reverse and forward transfected with specific mouse FGFR-1, FGFR-2 or control siRNA (Santa Cruz Biotechnology, sc-29317 and sc-29799). Transfected cells were then cultured in 0.5% serum-containing media for 72 hours; the cell number determined with a hematocytomer in quadruplicate and normalized to control siRNA condition.

*In vitro* lymphangiogenesis coculture assay was done using a modified version of Sakamoto’s method [5], [20]. Briefly, 500,000 tumor cells were mixed with 500 µl of collagen 1 (2 mg/ml, for C6 cells) or growth factor-reduced matrigel (BD Biosciences, for 66c14 cells) and plated in 24 or 48-well plates (for C6 and 66c14 respectively). HMVEC-dLys (150,000 and 15,000 cells for C6 and 66c14 experiments respectively) were seeded on the top of the matrix gel. Recombinant human VEGF-A and VEGF-C (20 and 100 ng/ml respectively) or VEGFR-2/Fc and VEGFR-3/Fc chimera (1 µg/ml) were added to the respective culture media. Lymphatic tube formation was monitored after 24 hours and lymphatic-like structures were visualized by prox-1 (Abcam, for C6 co-culture) or LEL (Lycopersicon Esculentum (Tomato) Lectin, Vector; for 66c14 co-culture) staining. Counterstaining was done with DAPI (Invitrogen). Stainings were visualized using a 100X or a 200X magnification (for respectively prox-1 and LEL staining) on a Leica confocal microscope.

For supernatant-induced lymphatic-like structure formation, 66c14 cells were plated at 10^6^ cells per dish (6 cm diameter) and cultured overnight. The media was then replaced with fresh basal media without serum and collected 24 hours later. Tube formation was then performed as described just above. Phase contrast images were taken with an Olympus FSX-100 microscope at 200X magnification.

For VEGF-C western blotting, 66c14 tumor cells were incubated in serum-free basal media, for 24 hours. The following day, cell supernatants were concentrated using a 10 kDa cut-off amicon concentrator column (Millipore), and the total protein concentration determined by BCA (Pierce). Equal amounts of supernatant proteins underwent SDS-PAGE, transferred on PVDF membrane and incubated with a Goat anti VEGF-C antibody (Santa Cruz Biotechnology, clone C20, sc-1881). Coomassie blue staining of the corresponding membrane was utilized as loading control.

**Figure 2:**
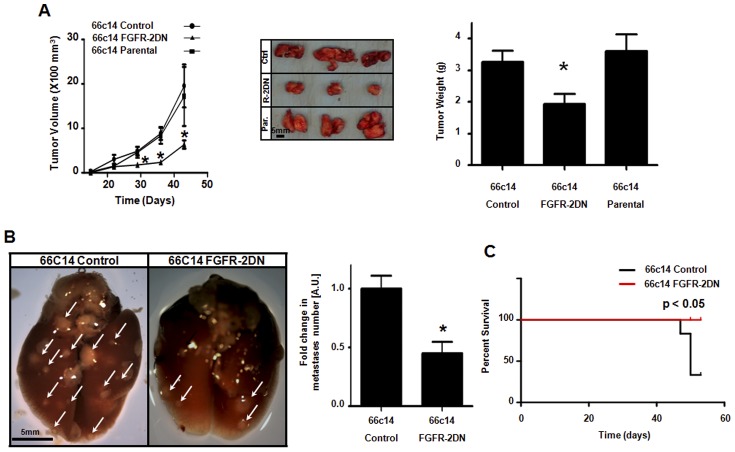
Inhibition of FGFR signaling suppresses primary tumor growth, metastasis and improves mouse overall survival. (A) Left panel, tumor growth is reduced in FGFR-2DN-expressing 66c14 carcinoma tumors (triangle) compared to parental (square) and empty plasmid-transfected (Control, circle) groups. Middle panel, representative images of 66c14 tumors confirm a decrease in tumor size in FGFR-2DN-expressing group (middle) compared to control (empty plasmid-transfected cells, upper) or parental (untransfected cells, lower). Right Panel, 66c14 tumor weight is reduced in FGFR-2DN group compared to control or parental group. (B) Left panel, representative images of lungs from mice injected with FGR-2DN-expressing 66c14 tumor cells (right) showing a decrease in metastatic nodules (indicated by white arrows) as compared to control group (empty plasmid-transfected cells, left). Right panel, a two-fold decrease in the number of metastatic nodules per lung is observed in mice bearing FGFR-2DN-expressing tumors compared to control group. (C) Survival is increased in mice bearing FGFR-2DN-expressing 66c14 tumors (red) versus control group (black). (Scale Bars, 5 mm in A and B, *p<0.05 versus respective control group).

For phospho-protein western blotting, 66c14 tumor cells were serum-starved overnight. The following day, cell treatment with the FGFR inhibitor PD-173074 (30 µM, for 10 or 60 minutes) was performed, in the presence or absence of FGF-2 (20 ng/ml, 10 minutes). Cells were lysed in RIPA buffer and the total protein concentration determined by Bradford (Biorad). Equal protein amounts underwent SDS-PAGE, transferred on nitrocellulose membrane and incubated with the PathScan Multiplex Western Cocktail I to detect phospho-p90RSK, phospho-Akt, phospho-p44/42 MAPK and phospho-S6 Ribosomal Protein (Cell Signaling Technology, #5301). Rab11 expression level, detected using the same antibody cocktail, was utilized as loading control, and kinase activities were normalized to their respective controls. Representative western blot and densitometry is shown.

**Figure 3:**
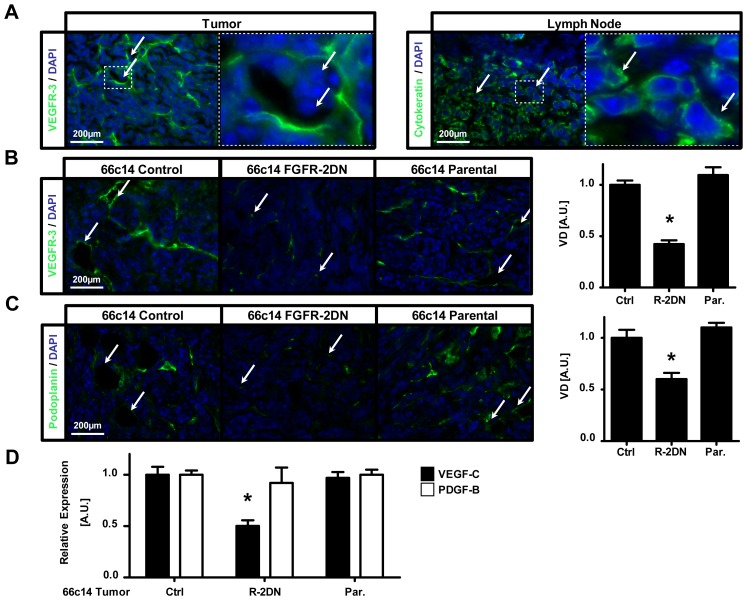
Inhibition of FGFR signaling suppresses tumor lymphangiogenesis and VEGF-C expression. (A) Left Panel, 66c14 tumor cells are observed into the lumens of VEGFR-3-positive lymphatic vessels (green) in 66c14 control tumors (white arrows in both left image and right zoomed-inset). Right panel, cytokeratin-stained 66c14 tumor cells (green) are detectable in axillary lymph nodes of 66c14 control cells-bearing mice (white arrows in both left image and right zoomed-inset), confirming the invasion mechanism via the lymphatic system of the 66c14 cells. (B) Left panel, representative images of VEGFR-3 (green) and DAPI (blue) staining of parental, empty plasmid (Control) and FGFR-2DN-expressing 66c14 tumors sections. White arrows indicate lumenized lymphatic vessels or isolated lymphatic endothelial cells in controls (Control and Parental) and FGFR-2DN tumors, respectively. Right panel, quantification of VEGFR-3-positive lymphatic vessel density (VD) demonstrates a density decrease in FGFR2-DN (R-2DN) expressing 66c14 tumor as compared to parental (Par.) or control (Ctrl) tumors. (C) Upper panel, FGFR-2DN-expressing 66c14 (66c14 FGFR-2DN) tumors exhibit a decrease in podoplanin-positive lymphatic vessel (green) density compared to control groups (66c14 Control and Parental). White arrows confirm the presence of lumenized lymphatic vessels or isolated lymphatic endothelial cells in controls and FGFR-2DN tumors, respectively. Bottom panel, quantification of podoplanin-positive lymphatic vessel density (VD) confirms a density decrease in FGFR2-DN (R-2DN) expressing 66c14 tumor as compared to parental (Par.) or control (Ctrl) tumors. (D) VEGF-C and PDGF-B (black and white bars, respectively) mRNA quantification of 66c14 tumor by qRT-PCR shows uniquely a VEGF-C expression decrease in 66c14 FGFR-2DN-expressing (R-2DN) versus control tumors (Ctrl; 66c14 control and Par; parental). (Scale Bars, 200 µm in A–C, *p<0.05 versus respective control groups).

### Standard and Quantitative RT-PCR

Total RNA was extracted from cells using the RNAEasy mini kit (Qiagen) under conditions recommended by manufacturer. RNA concentration was determined using a nanodrop spectrophotometer (Nanodrop-ThermoScientific) and 1 µg of total RNA was reverse transcribed using the SuperScript III First-Strand Synthesis kit (Invitrogen). Standard PCRs were performed using mouse FGFs and FGFRs-specific primers (Table S1), GoTaq DNA polymerase (Promega) on an eppendorff thermocycler for 35cycles. Mouse brain cDNA and water were used as positive and negative control, respectively.

Quantitative PCRs were performed using gene-specific primers (SABiosciences Corporation), SYBR Green mix (ABgene) on an ABI Prism 7900 HT Real-Time PCR System (Applied Biosystems) using a 384-well plate. Quantification was performed by the standard curve method using GAPDH gene as normalizer.

### Histology and Immunostaining

For C6 tumors, 10 µm sections were obtained using a microtome and stored at room temperature until use. Deparaffinized sections were treated with 10 mM Sodium Citrate in a microwave oven prior to immunostaining. For 66c14 tumors, 6 µm frozen tumor sections were air-dried for 30 minutes at room temperature and washed 3 times in PBS before immunostaining.

The following primary antibodies were then incubated overnight at +4°C: Goat anti mouse VEGFR-3 (R&D Systems, AF743), Syrian hamster monoclonal anti mouse podoplanin (Developmental Studies Hybridoma Bank, University of Iowa, 8.1.1 clone) and Rabbit anti cytokeratin (Dako, Z0622 ). Staining was then visualized by incubation with adapted secondary antibodies (Molecular Probes for fluorescent antibodies, DAKO for HRP-conjugated antibodies) and, if necessary, with DAB (DAKO). Pictures from at least five tumor areas were taken in the individual corresponding fluorescent channel using an Olympus IX71 inverted microscope at 400 X magnification. Peroxydase staining pictures were taken as previously described (Auguste et al, 2001). For each picture, vessel density was manually determined using the Image J software. For each experiment, specificity of the labeling was controlled by omitting the primary antibody (Figure S10).

### Statistical Analysis

Data are shown as mean ± Standard Error of the Mean (SEM) of 6 to 9 samples from 2 to 3 independent experiments. Statistical analyses were carried out using Statview (SAS Institute, Inc.) or GraphPad Prism (for the Kaplan-Meier survival curves). A P value less than 0.05 (*, #) was defined as statistically significant.

**Figure 4:**
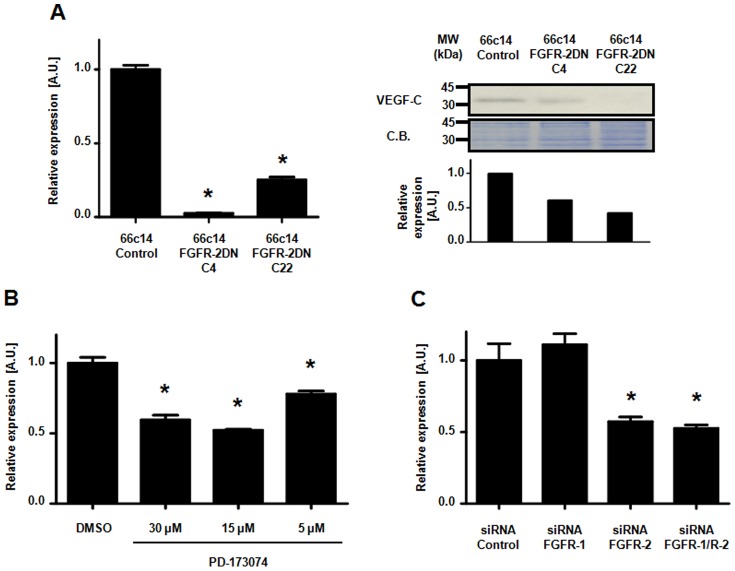
Blockade of Fibroblast Growth Factor Signaling suppresses VEGF-C expression in 66c14 cancer cells. (A) Left panel, decrease in VEGF-C mRNA expression is detected in FGFR-2DN-expressing 66c14 tumor cells (clones C4, C22) as compared to empty plasmid-transfected group (Control). Right panel, inhibition of VEGF-C protein secretion in FGFR-2DN-expressing 66c14 cell supernatant (C4 and C22) was confirmed by western blotting, and normalized to control group using coomassie blue (C.B.) staining of the membrane as loading control. (B) The FGFR inhibitor PD-173074 inhibits VEGF-C mRNA expression in a dose dependent manner in 66c14 tumor cells. (C) Specific siRNA-mediated inhibition of FGFR-2, but not of FGFR-1, expression reduces VEGF-C mRNA level in 66c14 tumor cells. (*p<0.05 versus respective control group).

## Results

### Inhibition of Fibroblast Growth Factor Activity Blocks Proliferation, Metastasis and Extends Survival in Tumor-bearing Mice

To investigate the role of FGFR signaling in tumorigenesis, metastasis and tumor lymphangiogenesis, we first inhibited FGFR signaling in mouse mammary carcinoma cells (66c14) using the dominant-negative FGF receptor strategy [4], [13], [14]. 66c14 cells are known to metastasize to the lungs mainly via the intratumor lymphatic vessels, in a VEGF-C-dependent manner, when injected directly into the mammary fat pad [18], [21]. Expression of multiple FGF ligands and receptors (including FGF-2, FGFR-1 and both FGFR-2 IIIb and IIIc isoforms) was also detected by RT-PCR in 66c14 tumor cells (Figure 1A and data not show for FGFR-2 isoforms). FGF-2, the FGF ligand prototype, was shown to bind with a high affinity to the IIIc splice variant of both FGFR-1 and R-2 [1], and the expression of a dominant negative form of this receptors splicing variant, truncated for its intracellular tyrosine kinase domains, was capable of disrupting FGF-2-mediated biological functions [4], [13], [14].

Three 66c14 clones stably transfected with the FGFR-2IIIc dominant negative construct (FGFR-2DN) were selected based on the analysis of mRNA expression by quantitative RT-PCR (66c14 FGFR-2DN C4, C18 and C22, Figure 1B and S1A) and their *in vitro* growth analyzed. A decrease in proliferation was observed when FGFR-2DN is expressed in the 66c14 cells (66c14 FGFR-2DN C4, C22, Figure 1D) compared to mock-transfected control cells. The FGFR inhibitor PD-173074 and FGFR-1 or FGFR-2 specific siRNA (Figure 1C) also reduced proliferation of 66c14 mock-transfected control cells (Figure 1E and 1F).

PD-173074-induced inhibition of 66c14 cell proliferation correlated with the respective up-and down-regulation of the cyclin inhibitor, p21, and the cyclin D1 mRNA expression (Figure S2A). C-Myc expression was increased in 66c14 cells treated with PD-173074. A decrease in both basal and FGF-2-stimulated Erk and S6 ribosomal protein phosphorylation was observed in PD-173074-treated cells (Figure S3A and B). A similar reduction in basal Erk activity was detected in FGFR-2DN-expressing 66c14 cells as compared to controls (Figure S4A and B). Interestingly, these mitogenic alterations might not be coupled to any modification in migration/invasiveness as no significant variation in epithelial-to-mesenchymal transition (EMT)-related or in metalloproteinase genes was detected, except for MMP-14, in FGFR inhibitor-treated 66c14 cells as compared to controls (Figure S2B).

Taken together, this data indicate that FGFR-2DN expression, FGFRs siRNA or treatment with the kinase inhibitor PD-173074 inhibits cancer cell proliferation, to a similar degree.

We next implanted dominant-negative or control 66c14 cells into the mammary fat pad of Balb/c mice to monitor *in vivo* growth of orthotopic mouse mammary tumors. As shown in Figure 2A, tumor development was slower in mice bearing FGFR-2DN-expressing tumors (left panel), leading to smaller tumors compared to mock-transfected and parental controls (middle and right panel).

66c14 carcinoma cells aggressively metastasize to the lungs (Figure 2B left panel, white arrows and [18]). We therefore evaluated metastasis in mice bearing FGFR-2DN-expressing tumors in comparison to control. As shown in Figure 2B, lungs of mice bearing FGFR-2DN-expressing 66c14 tumors exhibited a significant reduction in size and number of metastatic nodules (left panel). Quantification of foci number revealed a two-fold decrease in mice implanted with FGFR-2DN expressing cells compared to control (Figure 2B, right panel). As 66c14 tumors develop and disseminate in a tight and similar time lapse, mice bearing control tumors died in a narrow time period (50% mortality at 53 days post implantation, Figure 2C black line). Conversely, 100% of FGFR-2DN tumor-bearing mice survived at 53 days post implantation (Figure 2C, red line). Together, these results provide evidence that FGFR signaling modulates 66c14 tumor growth, dissemination of tumor cells to the lungs, and overall survival of mice.

**Figure 5:**
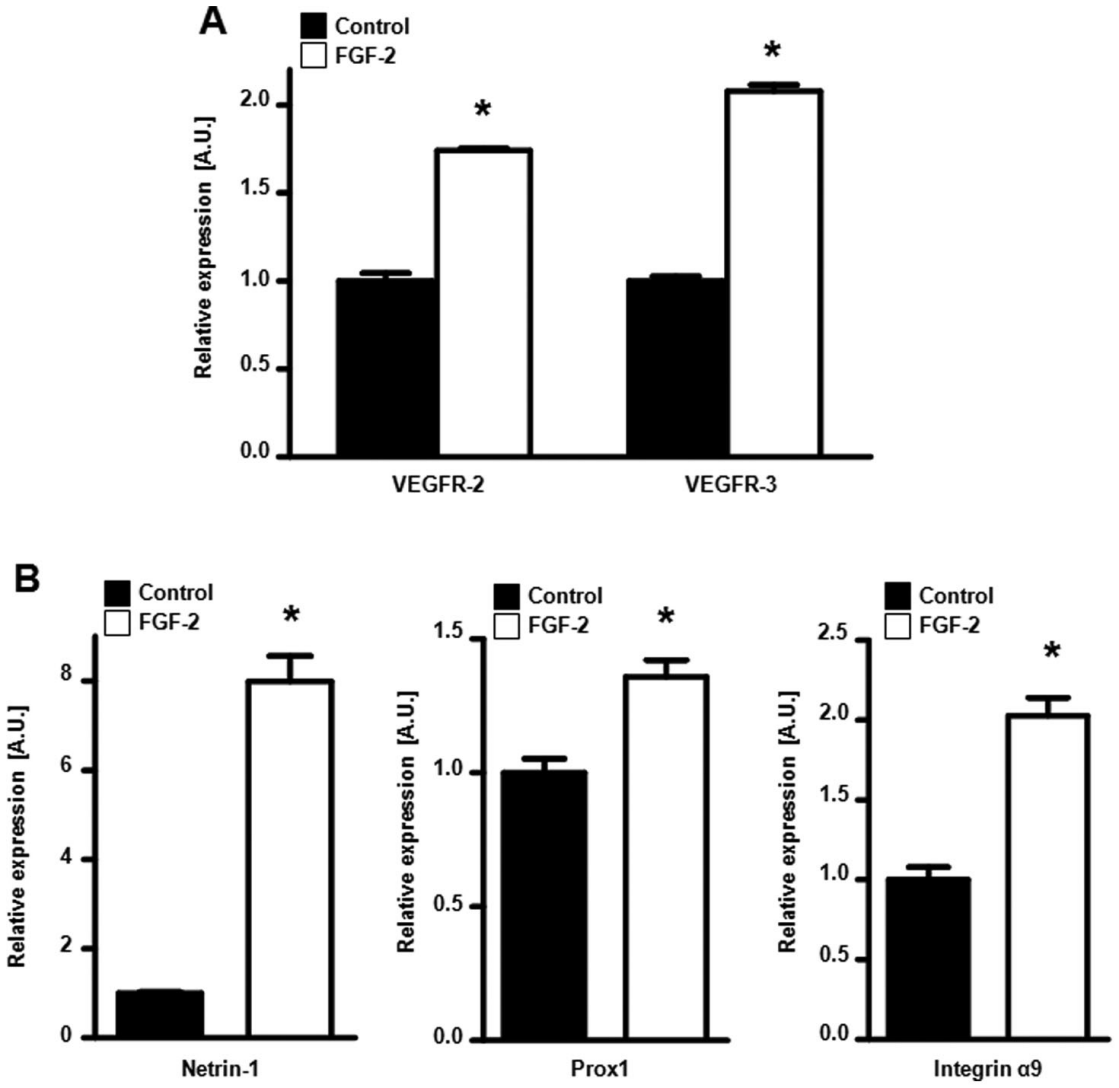
FGFR signaling stimulates expression of lymphangiogenic genes in lymphatic endothelial cells. (A) VEGFR-2 and VEGFR-3 mRNA expression is increased in FGF-2-treated (white) human dermal microvascular lymphatic endothelial cells (HMVEC-dLys) as compared to control (untreated cells, black). (B) Netrin-1 (left panel), Prox1 (middle panel) and integrin α9 (right panel) mRNA expression is stimulated by FGF-2 (white) in HMVEC-dLys as compared to control (untreated cells, black). (*p<0.05 versus respective control group).

### Inhibition of FGFR Signaling Suppresses Lymphangiogenesis in Primary Tumors by Reducing VEGF-C Expression in Tumor Cells

We next sought to explain the observed decrease in tumor metastasis. Development of a tumor lymphatic system has been shown to contribute to tumor dissemination [6]. Cancer cells intravasate into lymphatic vessel lumens, migrate through the lymphatic system, and successively invade lymph nodes and distal organs such as lungs [8], [9]. Thus, we first ascertained that 66c14 tumor cells could be detected in the lumen of VEGFR-3-positive tumor lymphatic vessels (Figure 3A left panel, white arrows) and that they invaded distal axillary lymph nodes (Figure 3A right panel, white arrows). These data are in agreement with previous findings by Caunt et al., who reported that inhibition of functional intratumoral lymphatic vessels, via the blockade of VEGF-C-induced cell functions, decreased metastasis of 66c14 cells to lymph nodes and lungs [18].

To determine whether the decrease in tumor metastasis was associated with modification of the lymphatic vessel density, tumor sections were stained with anti-VEGFR-3 or podoplanin antibodies. FGFR-2DN-expressing tumors displayed a decrease in lymphatic vessel density, compared to mock transfected control or parental groups, 6 weeks after the injection of tumor cells into the mammary fat pads (data not shown). To rule out the possibility that the vascular changes were caused by a difference in tumor size, we performed staining for lymphatic vessel markers on tumor tissue isolated 3 weeks after implantation, a time point where no significant growth difference was detected (Figure 2A). Once again, we detected a reduction in the density of VEGFR-3-positive lymphatic vessels in FGFR-2DN-expressing tumors as compared to mock-transfected or parental tumors (Figure 3B). As FGF signaling has been shown to regulate endothelial VEGFR-3 expression [10], to avoid any underestimation of lymphatic vessel in the FGFR-DN group, podoplanin staining was also performed on tumor sections 3 weeks after implantation. As expected, the density of tumor lymphatic vessel was reduced in FGFR-2DN tumors versus control groups (Figure 3C). Interestingly, a switch from lumenized vessels to an isolated endothelial cell (EC) phenotype was observed in FGFR-2DN-expressing tumors compared to control (Figure 3B and C, white arrows).

VEGF-C and PDGF-B are the most important factors implicated in tumor lymphangiogenesis [8], [22]. In our 66c14 tumor model, the vascular phenotype correlated only with an inhibition of VEGF-C mRNA expression in FGFR-2DN-expressing 66c14 compared to control tumors (Figure 3D). This indicates that VEGF-C but not PDGF-B is an intermediate of FGF-induced tumor lymphangiogenesis. Caunt and coworkers also demonstrated that subcutaneous rat C6 glioblastoma tumors develop a VEGF-C-dependent tumoral lymphatic vessel network [18], while no lymphangiogenesis is normally observed *in situ*. To validate our results in an independent tumor model, FGFR-2DN or mock transfected C6 tumor cells were subcutaneously injected in immunodeficient mice as previously described [4]. A decrease in the density of VEGFR-3 and podoplanin-positive lymphatic vessels, an increase in the ratio of isolated ECs to structured vessels (Figure S5A red arrows and data not shown), and a reduction in VEGF-C mRNA expression (Figure S5B) were also detected in FGFR-2DN-expressing C6 tumors (2A7 and C18) versus the control group (BH2). These data provide evidence that FGFR signaling promotes tumor lymphangiogenesis *in vivo* by regulating VEGF-C expression and EC organization in functional lumenized vessels.

**Figure 6:**
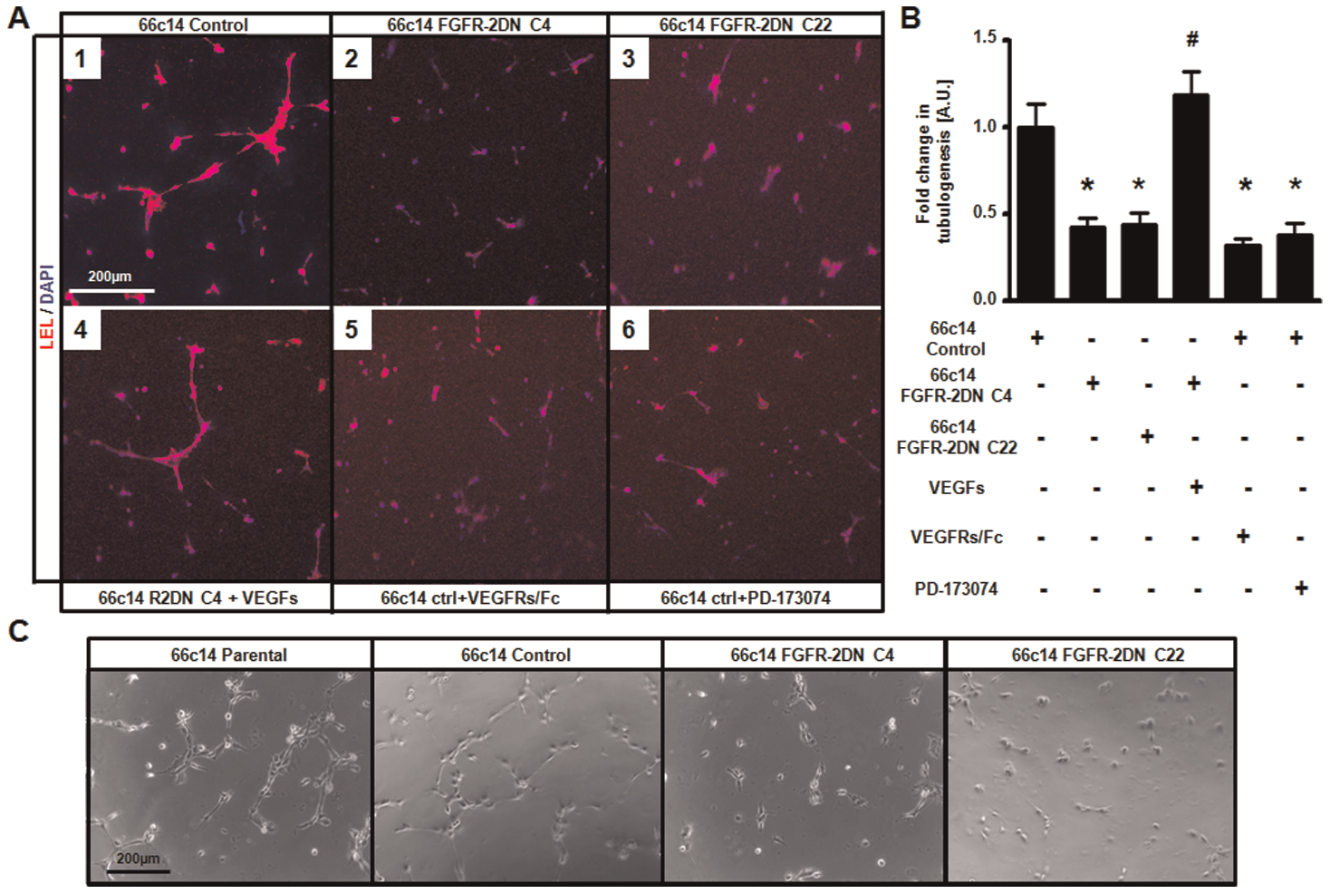
*In vitro* lymphangiogenesis is inhibited by FGFR-2DN-expressing 66c14 tumor cells. (**A**) Co-culture of human lymphatic endothelial cells and mouse mammary 66c14 carcinoma tumor cells induces lymphatic vessel-like tubes in the presence of 66c14 control cells (A1) and FGFR-2DN clone C4-expressing cells in combination with VEGFs (A4) while no vascular structure is observed in FGFR-2DN clone C4 (A2) or clone C22-expressing cells (A3), control cells pretreated with VEGFRs/Fc chimera (A5) or with FGFR inhibitor PD-173074 (A6). (B) Lymphatic tube formation was quantified and expressed as fold change as compared to control condition (A1). (C) *In vitro* lymphatic tubes formation was induced in the presence of supernatant from control or parental, but not from FGFR-2DN C4 and C22-expressing 66c14 carcinoma cells. (Scale bars, 200 µm in A and C, # and *p<0.05 versus respective control group).

### Blockade of Fibroblast Growth Factor Signaling Suppresses VEGF-C Expression in Tumor Cells

We next investigated whether FGFR-DN expression, FGFRs siRNA or PD 173074 modifies VEGF-C expression in the cancer cells. VEGF-C mRNA expression was measured in FGFR2-DN-expressing, FGFRs siRNA or PD 173074-treated cells. In 66c14 cells (Figure 4A left panel and S1B), expression of FGFR-2DN led to a strong reduction in VEGF-C mRNA level, when compared to mock-transfected cells. This decrease was confirmed at the protein level, in the concentrated supernatant of FGFR-2DN-expressing 66c14 cells compared to control cells (Fig 4A right panel and S1C). PD-173074 (5–30 µM) and FGFR-2, but not FGFR-1, siRNA also down-regulated VEGF-C mRNA expression (Figure 4B and C; respectively). Conversely, FGF-2-treated 66c14 cells displayed an increased VEGF-C mRNA expression (Figure **S6**). In C6 cells, a decrease in VEGF-C was also detected in FGFR-2DN- transfected cells (clones 2A7, C18) in comparison to control (BH2, Figure S7A).

Our data clearly demonstrate that FGFR signaling positively regulates VEGF-C expression in 66c14 and C6 cancer cell types and that FGFR blockade inhibits this.

### FGFR Signaling Stimulates VEGF-C Expression Independently of Either COX-2, HIF-1α or NF-κB

Both cyclooxygenase-2 (COX-2) and hypoxia inducible factor-1α (HIF-1α) have been reported to modulate VEGF-C expression in different tumor models [15], [16], [17]. FGFR-2DN-expresssing 66c14 cells displayed a decrease in COX-2 and HIF-1α mRNA expression levels, when compared to mock-transfected cells (Figure S8A). Decreased mRNA levels were also seen when cells were treated with PD-173074 (Figure S8B). However, inhibition of COX-2 or HIF-1α in 66c14 cells using specific inhibitors (5–100 µM of NS-398 and 30–100 µM of #400083, inhibitors of Cox2 and HIF-1α, respectively) was not sufficient to modify VEGF-C mRNA expression level in either normoxia or cobalt chloride-induced hypoxia (Figure S8C and D, respectively). However, changes in VEGF-A expression were detected, validating the cobalt-induced hypoxia and inhibitor efficacy. Similarly, chemical inhibition of NF-κB, another known regulator of VEGFs, did also not alter VEGF-C expression (0.1 to 5 µM, Cayman chemical #CAY10512, data not shown). As expected, NS-398 had no effect on VEGF-C mRNA expression in C6 cells either (Figure S7B). These data clearly show that VEGF-C expression is regulated by FGFs through a mechanism other than simple regulation of COX-2, HIF-1α and NF-κB.

### FGFR Signaling Stimulates Expression of Lymphangiogenic Factors in Endothelial Cells

We next investigated whether tumor-derived FGFs could act in a paracrine manner on lymphatic endothelial cells. We examined the expression of selected lymphangiogenic genes in HMVEC-dLys treated with FGF-2 for 48 hours. We found that activation of FGFR signaling in HMVEC-dLys led to an increase in the expression level of VEGF receptors (VEGFR-2 and VEGFR-3), netrin-1, prox1 and integrin α9 (Figure 5A–B). These data further demonstrate that FGFR signaling acts on lymphatic endothelial cells to enhance the lymphangiogenic response.

### *In vitro* Lymphangiogenesis is Inhibited by Expression of FGFR-2DN in Tumor Cells

We next examined whether inhibition of FGF signaling in tumor cells is sufficient to explain the reduction in tumor lymphangiogenesis using an *in vitro* lymphangiogenesis co-culture assay [5], [20]. Briefly, FGFR-2DN expressing 66c14 and C6 cells or empty vector transfected control cells, were mixed with growth factor-reduced matrigel or collagen-1, respectively, and added onto tissue-culture plates. After polymerization, HMVEC-dLys were seeded on the top of the tumor cell-containing gels. Lymphatic tube formation was assessed by LEL or prox1 staining (for 66c14 and C6 cocultures, respectively) and confocal microscopy. As expected, lymphatic tubes were observed when gels contained mock-transfected cells (Figure 6A1, B and S9A), while isolated unorganized lymphatic EC clusters were observed in the presence of FGFR-2DN-expressing cells (Figure 6A2;3, B and 9B-CS). The same result was obtained when control cells were treated with PD-173074 (Figure 6A6 and B).

To prove that lymphatic tubulogenesis depended on endothelial soluble factors, rather than a cell-cell contact mechanism, lymphatic endothelial cells, plated on matrigel, were incubated with the supernatants from FGFR-2DN C4-, C22-expressing, control or parental 66c14 cells (Figure 6C). Lymphatic endothelial cells formed vascular tubes upon treatment with parental and control cells supernatants, while isolated lymphatic ECs were observed in the FGFR-2DN-conditioned media groups (Figure 6C).

To demonstrate the VEGF dependency, VEGF-A and VEGF-C recombinant proteins were supplemented into the gel with FGFR2-DN-expressing 66c14 cells. VEGF addition rescued the loss of lymphatic tube formation (Figure 6A4 and B). On the other hand, mock-transfected 66c14 cells mixed into the gel with VEGFR-2/Fc and VEGFR-3/Fc chimeras (Figure 6A5 and B), led to an inhibition of tubulogenesis (as compared to Figure 6A1;5 and B). Finally, blood vascular endothelial cells (human dermal microvascular endothelial cells, HMVEC-ds), which endogenously secrete VEGF-A and VEGF-C, were seeded instead of FGFR-2DN-expressing cells. This stimulated the formation of capillary-like structures (Figure S9D).

Together, these data indicate that FGFR signaling stimulates, in both tumor cell lines *in vitro* lymphatic vessel formation, through VEGF-C.

**Figure 7:**
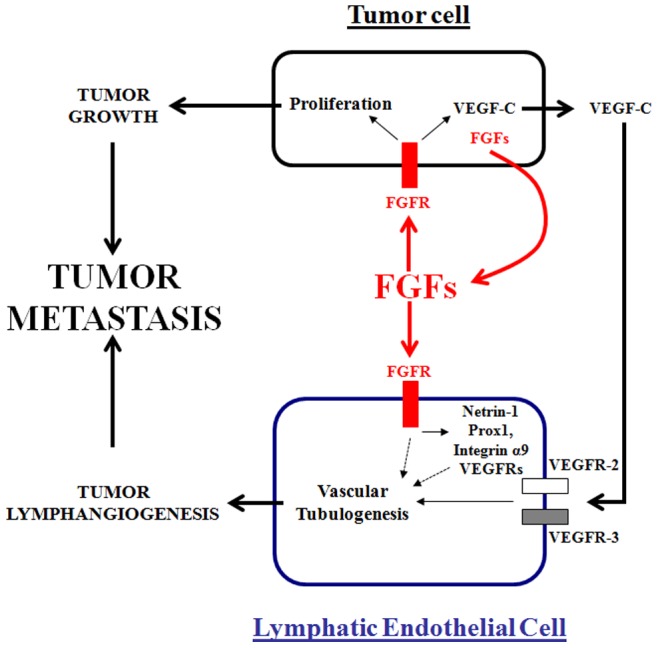
Schematic representation of FGFs-mediated tumor growth, metastasis and lymphangiogenesis. Tumor-secreted FGFs (red) play a central role in the induction of tumor metastasis, both directly by stimulating cancer cell proliferation and indirectly by upregulating VEGF-C expression in tumor cells (black). Tumor secreted FGFs might also induce directly lymphatic tube formation as previously demonstrated *in vitro* (dashed black line). Thus, tumor VEGF-C activates its VEGFR-2 and VEGFR-3 receptors on lymphatic endothelial cells, leading to lymphatic vessel formation. Tumor FGFs promote also pro-lymphatic gene expression (such as VEGFR-2, VEGFR-3, netrin-1, prox1 and integrin α9) in lymphatic endothelial cells (blue). Both tumor growth and lymphangiogenesis lead to tumor metastasis.

## Discussion

Fibroblast growth factors are expressed in a number of tumors and have regulatory functions in tumor development. Thus, targeting FGFR signaling may hold promise for therapy [1]. However, attention has been mostly focused on other receptor tyrosine kinase signaling pathways, such as VEGF receptors, so far, resulting in an incomplete study of the role of FGFR signaling in tumor progression and dissemination.

We demonstrate herein that inhibition of FGFR signaling not only impairs tumor growth *in vitro* and *in vivo*, but also affects lymphangiogenesis and metastasis. As previously reported, we confirm the existence of an autocrine FGF-loop in tumorigenesis [4], [23]. The intracellular effector Erk is activated by FGFR signaling, and its inhibition correlated with an impairment of cancer growth [1], an increased p21 and a decreased cyclin D1 expression [24]. Similarly, up-regulation of the proto-oncogene *c-myc,* upon inhibition of FGFR signaling, might reflect an arrest in cell growth and an induction of cell apoptosis [25].

A reduction in lung metastatic foci size and number was also observed in mice implanted with FGFR-2DN cells. Cancer cells need to leave the primary tumor site and to invade distant organs. Taeger et al. reported that inhibition of FGFR signaling results in a decrease in tumor cell motility due to reduction in integrins, extracellular matrix proteins, mediators of epithelial-mesenchymal transition, and MMPs [23], [26]. However, we observed that FGFR signaling blockade, in 66c14 carcinoma cells, did not alter significantly expression of EMT and invasion markers, except for MMP-14, confirming the role of FGF family molecules in its regulation [27].

Other evidence over the past 15 years also clearly reveals the crucial role of the tumor lymphatic system, and its main inducer, VEGF-C, in metastasis [8]. However it is not yet known whether FGFR signaling has a role in tumor lymphangiogenesis. We demonstrate herein that FGFR signaling indeed plays a role in tumor lymphangiogenesis. Inhibition of FGF activity in tumor cells abrogated tumor lymphangiogenesis *in vitro* and *in vivo* by down-regulating VEGF-C expression, in two independent tumor models. To our knowledge, this is the first demonstration that, in two different tumor settings, FGFR signaling modulates tumor lymphangiogenesis.

In addition to this autocrine mechanism on tumor cells, FGF acts directly on lymphatics by modulating lymphangiogenesis genes. We reported that loss of FGF signaling led, *in vitro* and *in vivo*, to a disruption of lymphatic vessel morphogenesis and an increase in the number of isolated endothelial cells. This was rescued *in vitro* by exogenous VEGFs. Interestingly, a similar phenotype was seen in FGFR-DN-expressing (blood) vascular endothelial cells that down-regulate both VEGFR-2 expression and response to VEGF-A stimulation [14]. Finally, it has been demonstrated that both FGFR and VEGFR signaling is necessary, to promote EC migration and tubular morphogenesis [28]. Taken together, these results emphasize the idea of an interconnection or synergy between the FGF and VEGF pathways. Onimaru et al. described a similar lymphangiogenic connection between FGF and PDGF-B [29]. However, inhibition of FGFR signaling in 66c14 tumor cells did not alter PDGF-B expression level *in vitro* or *in vivo*.

We also observed that the down-regulation of VEGF-C was associated with a decrease in both COX-2 and HIF-1α mRNA expression. COX-2 has been shown to induce lymphangiogenesis in human breast cancer via an upregulation of VEGF-C [15]. However, in our models, COX-2 inhibitors did not modulate VEGF-C expression in tumor cells; *in vitro*, similar to what has been previously reported [30]. It is likely that COX-2 activates lymphangiogenesis indirectly through other mechanisms, such as the upregulation of VEGF-C in macrophages [31]. Alternatively, VEGF-C might function as an upstream regulator of COX-2, leading to their successive inhibition upon FGFR inhibition [32]. HIF-1α is a well-studied inducer of VEGF-A ligand and receptor expression [33]. Several publications have also reported that VEGF-C expression correlates with HIF-1α levels [16], [17]. In our hands, inhibition of HIF-1α did not suppress VEGF-C expression, although a decrease in VEGF-A expression was detected. Further investigations are needed to completely elucidate the mechanisms controlling the induction of VEGF-C by FGFs.

It has been shown that the lymphangiogenic activity of FGF-2 is mediated, *in vitro* by the Akt/mTOR/p70S6 kinase pathway [11]. Furthermore, inhibition of pancreatic tumor lymphangiogenesis and metastasis has been achieved by rapamycin, a specific inhibitor of mTOR, which down-regulates VEGF-C [34]. As we reported that blockade of FGFR signaling reduces mTOR/p70S6 kinase pathway in 66c14, the potential link between FGFR signaling and mTOR in the regulation of VEGF-C expression needs further studies.

Our findings that FGFR signaling induces lymphangiogenic molecules in lymphatic endothelial cells, including VEGFR-2, VEGFR-3, netrin-1 and integrin α9, also validate the idea of FGF acting in a paracrine manner on tumor lymphatic endothelial cells. This strengthens the positive role of FGFs in tumor progression and dissemination.

In agreement with our results, Murakani and coworkers have recently demonstrated that dominant negative FGFR-expressing blood endothelial cells down-regulate VEGFR-2 expression, leading to a loss of response to VEGF stimulation *in vitro* and *in vivo* [14].

We also demonstrated that FGF signaling induces netrin-1 in lymphatic endothelium. Despite controversial data in the vasculature [35], [36], [37], compelling evidences support that netrins are pro- lymphangiogenic factors *in vitro* and *in vivo* [19], [38] and data not shown. Moreover, it has been shown that netrin-1, through its canonical receptors stimulates tumor growth and metastasis [39], [40]. Netrin-1 is highly expressed in metastatic human breast and pancreatic tumors and its inhibition by siRNA or soluble receptor strategies suppresses metastasis formation [39], [40].

Additional evidence indicates an important role for integrins in lymphangiogenesis and tumor metastasis [41], [42], [43], [44]. Integrin α9 expression is regulated in lymphatic endothelium by prox1. This integrin is required for lymphatic endothelial cell migration *in vitro* and development of a murine lymphatic system *in vivo* [45], [46]. Finally, direct interactions between lymphatic growth factors and integrins have been described, further supporting their role in lymphangiogenesis [47].

Taken together, these results confirm an important role for FGFR signaling in promoting tumorigenesis (Figure 7). FGFR signaling has pleiotropic effects on tumor development including (1) control of tumor growth, (2) promotion of tumor metastasis, (3) stimulation of tumor lymphangiogenesis, and (4) induction of VEGF-C and expression of VEGFRs or other pro-lymphangiogenic/survival factors in tumor cells and the lymphatic endothelium. Thus, antagonizing FGFR activity may be an interesting approach for anti-cancer therapy and not only limit primary tumor growth but also tumor lymphangiogenesis and metastatic spread.

## Supporting Information

Figure S1 **FGFR-2DN expression in 66c14 cells correlates with inhibition of VEGF-C mRNA and protein expression.** (A). FGFR-2DN mRNA is detected in FGFR-2DN-expressing 66c14 clone C18 cells but not in the empty vector-transfected control cells. (B) VEGF-C mRNA expression is decreased in FGFR-2DN-expressing 66c14 clone C18 cells versus mock-tranfected cells (Control). (C) A lower amount of VEGF-C protein is detected by western blotting in the supernatant of FGFR-2DN-expressing 66c14 clone C18 compared to control cells. Quantification was performed using coomassie blue (C.B.) staining of the membrane as loading control. (*p<0.05 versus respective control group).
(TIF)

Figure S2 **Expression of mitogenic, but not epithelial-to-mesenchymal transition or invasion markers is changed in 66c14 carcinoma cells upon FGFR signaling inhibition.** 66c14 were treated with the FGFR inhibitor, PD-173074 (30 µM) and mRNA expression level of markers of cell proliferation (A) or epithelial-to-mesenchymal transition (EMT) and invasion (B) was determined by quantitative RT-PCR. (*p<0.05 versus respective control group).
(TIF)

Figure S3 **Blockade of FGFR signaling inhibits basal and FGF-2-induced Erk and S6 ribosomal protein phosphorylation in 66c14 carcinoma cells.** Control 66c14 carcinoma cells were incubated with the FGFR inhibitor PD-173074 (30 µM) for 10 or 60 minutes, in the presence or absence of FGF-2 (20 ng/ml, for 10 minutes). Cell lysates were analyzed by western-blotting (A) to determine Erk, S6 kinase (B) and Akt, p90 RSK activation level (C). Rab11 expression level was used as loading control and kinase activities were normalized to their respective controls.
(TIF)

Figure S4 **Inhibition of basal Erk phosphorylation in FGFR-2DN-expressing 66c14 carcinoma cells.** Protein lysates of FGFR-2DN-expressing (C4, C18 and C22), control and parental 66c14 carcinoma cells were analyzed by western-blotting (A) to determine Akt, Erk, S6 kinase and p90 RSK activation level (B). Rab11 expression level was utilized as loading control and kinase activities were normalized to their respective controls.
(TIF)

Figure S5 **Inhibition of FGFR signaling suppresses C6 tumor lymphangiogenesis and VEGF-C expression.** (A) Upper panel, representative images of VEGFR-3 staining of control (BH2) or FGFR-2DN expressing (2A7 and C18) C6 glioblastoma tumor sections. Red arrows confirm the presence of lumenized lymphatic vessels or isolated lymphatic endothelial cells in controls and FGFR-2DN tumors, respectively. Bottom panel, quantification of VEGFR-3-positive lymphatic vessels shows a decrease in FGFR-2DN expressing C6 tumors (2A7 and C18) as compared to control tumors (BH2). (B) C6 tumor VEGF-C mRNA quantification by qRT-PCR shows an expression decrease in FGFR-2DN (2A7 and C18)-expressing versus respective control (BH2). (Scale Bars, 200 µm in A, *p<0.05 versus respective control group).
(TIF)

Figure S6 **FGF-2 induces VEGF-C mRNA expression in 66c14 cancer cells.** 66c14 cancer cells were incubated in the presence or the absence of recombinant FGF-2 (20 ng/ml) for different time durations, and total RNA retro-transcribed. VEGF-C mRNA expression was determined by quantitative PCR and expressed as fold change over control condition (red dashed line). (*p<0.05 versus control group, 0 h).
(TIF)

Figure S7 **In C6 cancer cells, blockade of Fibroblast Growth Factor Signaling suppresses COX-2 independent VEGF-C expression.** (A) VEGF-C mRNA expression is inhibited in rat C6 glioblastoma tumor cells expressing the FGFR-2DN (clones 2A7, C18) as compared to empty plasmid transfected control (control clone BH2). (B) VEGF-C mRNA expression is unchanged in C6 tumor cells treated with increasing doses of the COX-2 inhibitor NS-398. (*p<0.05 versus respective control group).
(TIF)

Figure S8 **FGFR signaling stimulates VEGF-C expression independently of either COX-2 or HIF-1α.** (A) COX-2 (black) and HIF-1α (white) mRNA expression is inhibited in FGFR-2DN-expressing 66c14 cells (clones C4, C18 and C22) compared to empty plasmid-transfected cells (control). (B) COX-2 (black) and HIF-1α (white) mRNA expression is inhibited in 66c14 cells treated with increasing doses of FGFR inhibitor PD-173074. (C) Left panel, VEGF-A (black) but not VEGF-C (white) mRNA expression is modified in 66c14 tumor cells treated with increasing doses of the COX-2 inhibitor NS-398. Right panel, the HIF-1α inhibitor 400083 decreases VEGF-A (black) but not VEGF-C (white) mRNA expression in 66c14 tumor cells, in normoxic conditions. (D) 66c14 cells were treated in the presence (+) or absence (-) of hypoxia inducer, cobalt chloride (10 mM), supplemented or not with various doses of cycloxygenase-2 (NS-398) or HIF-1α (400083) inhibitors (both concentrations in µM). VEGF-A (black) and VEGF-C (white) mRNA expression was then determined by qRT-PCR. VEGF-A, but not VEGF-C, hypoxia-induced mRNA expression was modified by both inhibitor treatments. (A–C: *p<0.05 versus respective control group; D: * and # p<0.05 versus cobalt chloride untreated and treated cells, without inhibitor, respectively).
(TIF)

Figure S9 **Expression of FGFR-2DN inhibits C6 cells-induced** ***in vitro*** **lymphangiogenesis.** Prox-1-stained lymphatic-like vascular tubes are observed in the co-culture between lymphatic endothelial cells (HMVEC-dLys) and C6 control (BH2, A) or blood endothelial cells (HMVEC-d, D), while unorganized lymphatic endothelial cell clusters are detected in co-culture with FGFR-2DN-expressing C6 cells (2A7 and C18, B and C, respectively).
(TIF)

Figure S10 **Immunohistochemical controls.** Immunohistochemical labeling of 66c14 tumor controls observed in the absence of VEGFR-3 (left panel), Podoplanin (middle panel) or Cytokeratin (right panel) primary antibody.
(TIF)

Table S1 **Mouse primer sequences for standard and quantitative RT-PCRs.**
(TIF)
