# Supplementary figures and images for: Blocking Fibroblast Growth Factor Receptor Signaling Inhibits Tumor Growth, Lymphangiogenesis, and Metastasis
Source: PLoS One. 2012 Jun 25;7(6):e39540. doi: 10.1371/journal.pone.0039540 (PMC3382584; doi:10.1371/journal.pone.0039540)

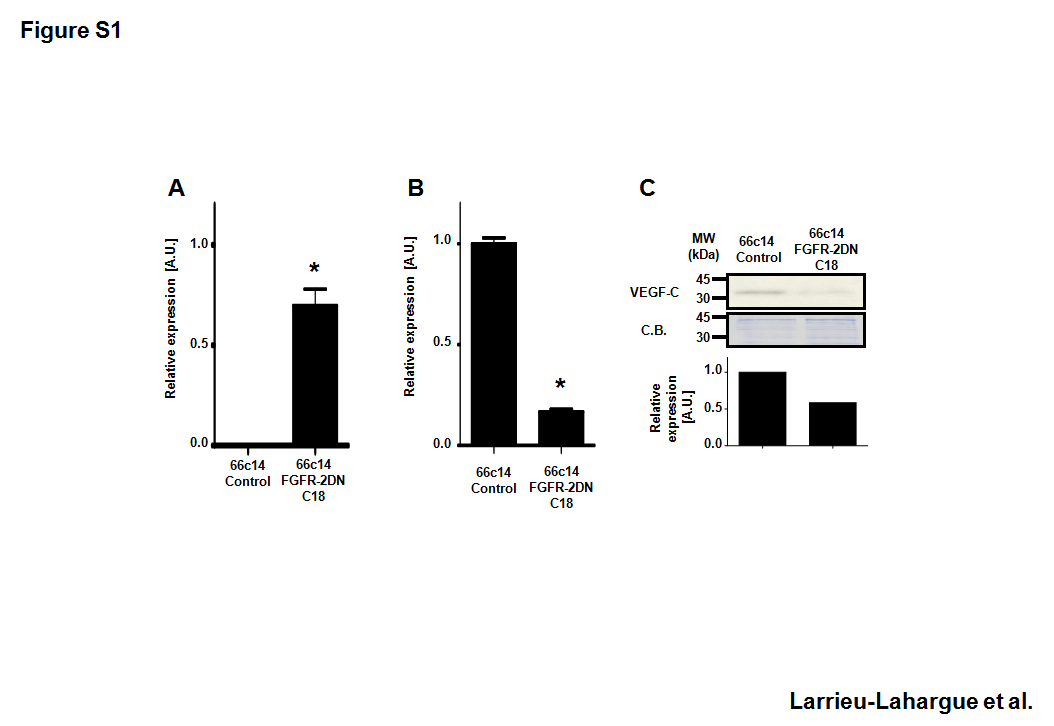

Supplement: Figure S1 — FGFR-2DN expression in 66c14 cells correlates with inhibition of VEGF-C mRNA and protein expression. (A). FGFR-2DN mRNA is detected in FGFR-2DN-expressing 66c14 clone C18 cells but not in the empty vector-transfected control cells. (B) VEGF-C mRNA expression is decreased in FGFR-2DN-expressing 66c14 clone C18 cells versus mock-tranfected cells (Control). (C) A lower amount of VEGF-C protein is detected by western blotting in the supernatant of FGFR-2DN-expressing 66c14 clone C18 compared to control cells. Quantification was performed using coomassie blue (C.B.) staining of the membrane as loading control. (*p<0.05 versus respective control group). (TIF) [file pone.0039540.s001.tif]

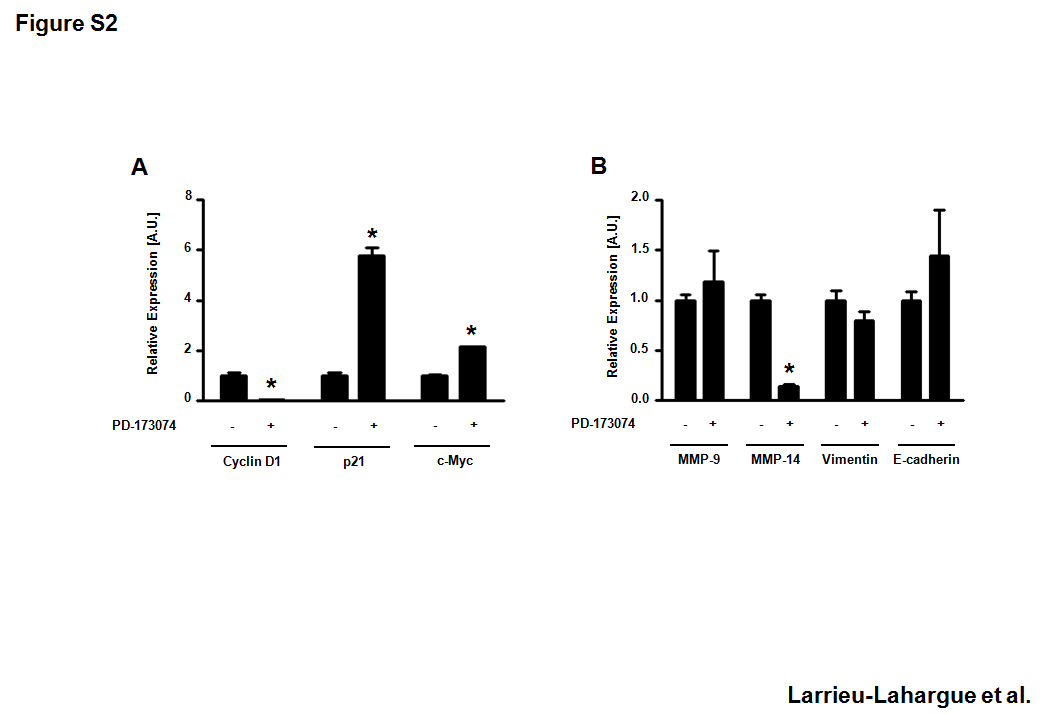

Supplement: Figure S2 — Expression of mitogenic, but not epithelial-to-mesenchymal transition or invasion markers is changed in 66c14 carcinoma cells upon FGFR signaling inhibition. 66c14 were treated with the FGFR inhibitor, PD-173074 (30 µM) and mRNA expression level of markers of cell proliferation (A) or epithelial-to-mesenchymal transition (EMT) and invasion (B) was determined by quantitative RT-PCR. (*p<0.05 versus respective control group). (TIF) [file pone.0039540.s002.tif]

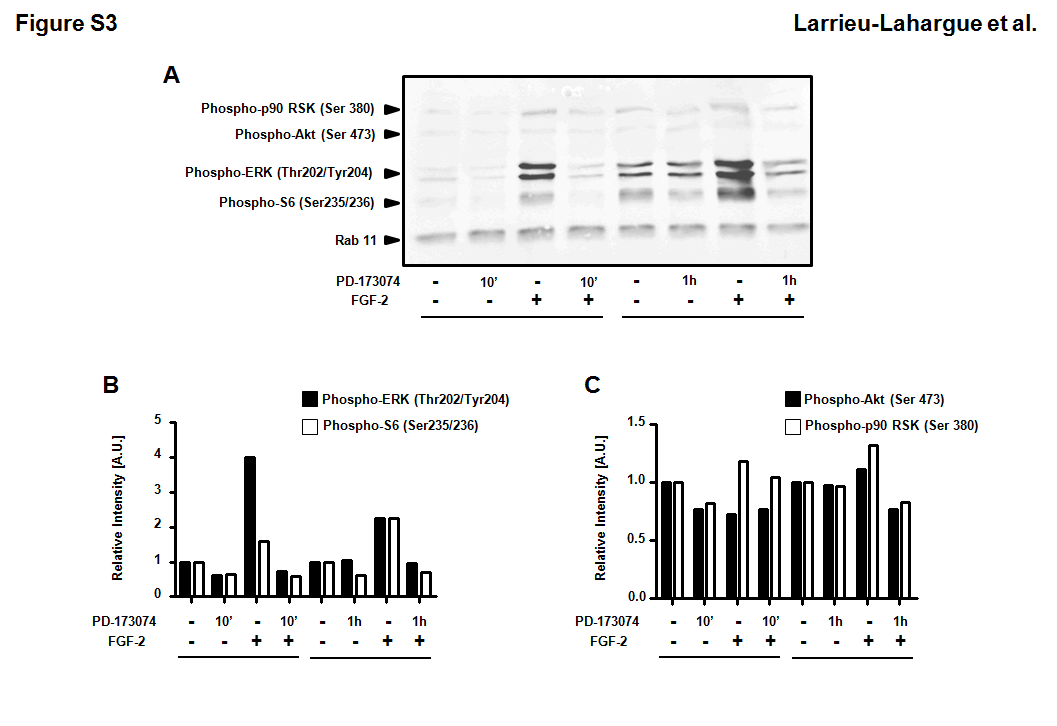

Supplement: Figure S3 — Blockade of FGFR signaling inhibits basal and FGF-2-induced Erk and S6 ribosomal protein phosphorylation in 66c14 carcinoma cells. Control 66c14 carcinoma cells were incubated with the FGFR inhibitor PD-173074 (30 µM) for 10 or 60 minutes, in the presence or absence of FGF-2 (20 ng/ml, for 10 minutes). Cell lysates were analyzed by western-blotting (A) to determine Erk, S6 kinase (B) and Akt, p90 RSK activation level (C). Rab11 expression level was used as loading control and kinase activities were normalized to their respective controls. (TIF) [file pone.0039540.s003.tif]

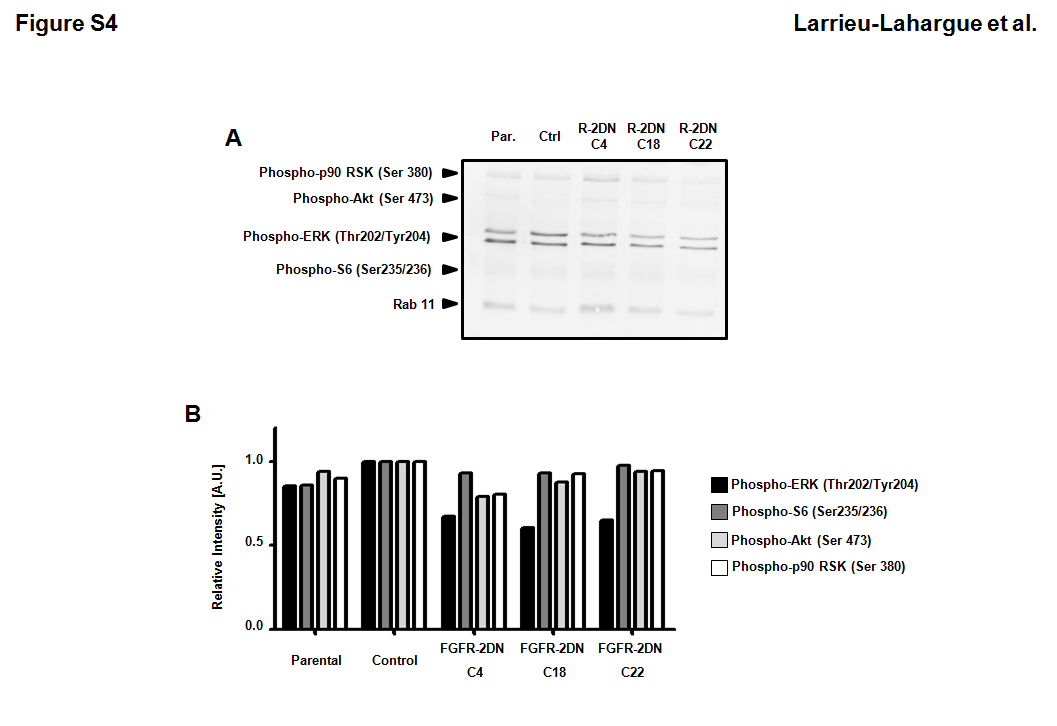

Supplement: Figure S4 — Inhibition of basal Erk phosphorylation in FGFR-2DN-expressing 66c14 carcinoma cells. Protein lysates of FGFR-2DN-expressing (C4, C18 and C22), control and parental 66c14 carcinoma cells were analyzed by western-blotting (A) to determine Akt, Erk, S6 kinase and p90 RSK activation level (B). Rab11 expression level was utilized as loading control and kinase activities were normalized to their respective controls. (TIF) [file pone.0039540.s004.tif]

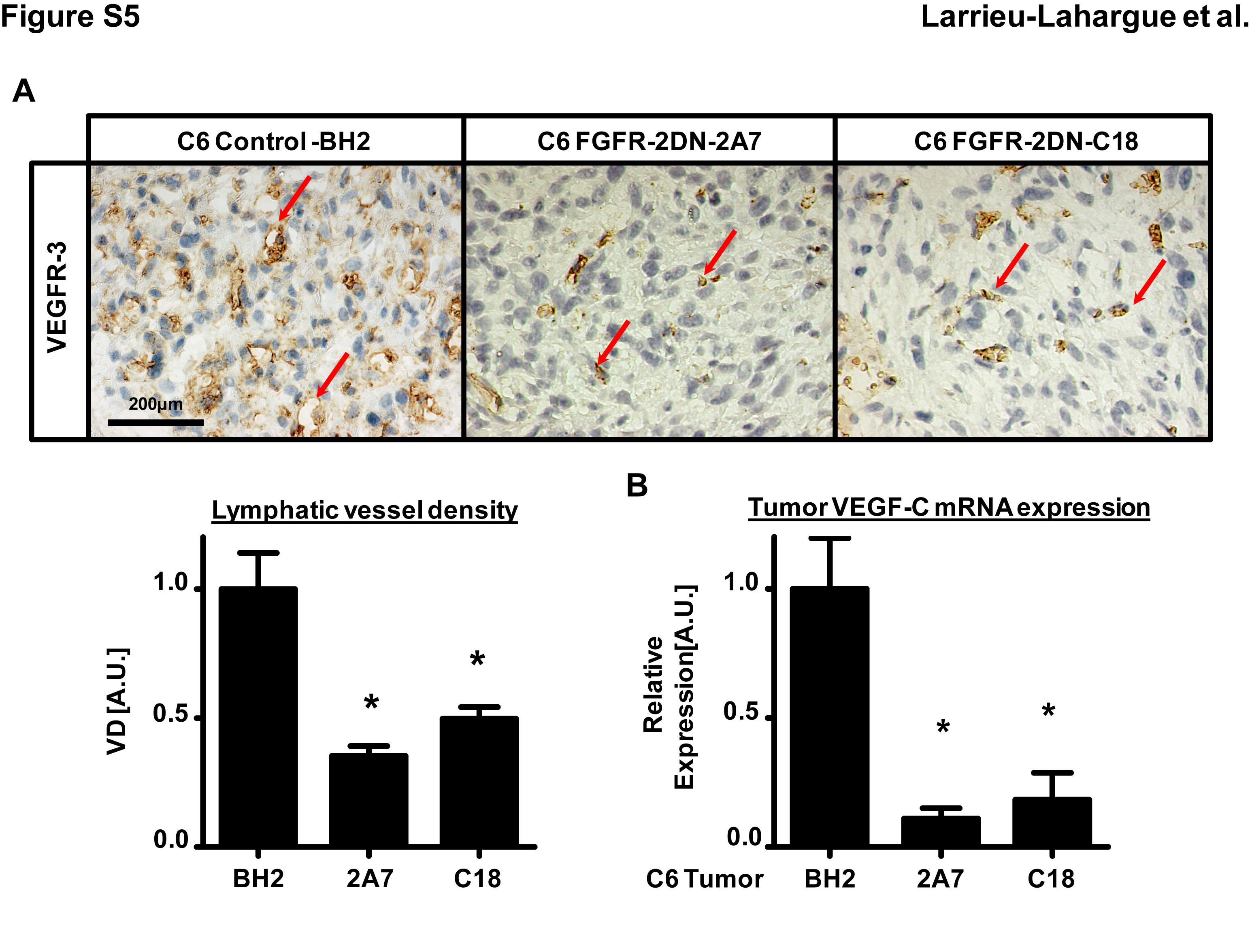

Supplement: Figure S5 — Inhibition of FGFR signaling suppresses C6 tumor lymphangiogenesis and VEGF-C expression. (A) Upper panel, representative images of VEGFR-3 staining of control (BH2) or FGFR-2DN expressing (2A7 and C18) C6 glioblastoma tumor sections. Red arrows confirm the presence of lumenized lymphatic vessels or isolated lymphatic endothelial cells in controls and FGFR-2DN tumors, respectively. Bottom panel, quantification of VEGFR-3-positive lymphatic vessels shows a decrease in FGFR-2DN expressing C6 tumors (2A7 and C18) as compared to control tumors (BH2). (B) C6 tumor VEGF-C mRNA quantification by qRT-PCR shows an expression decrease in FGFR-2DN (2A7 and C18)-expressing versus respective control (BH2). (Scale Bars, 200 µm in A, *p<0.05 versus respective control group). (TIF) [file pone.0039540.s005.tif]

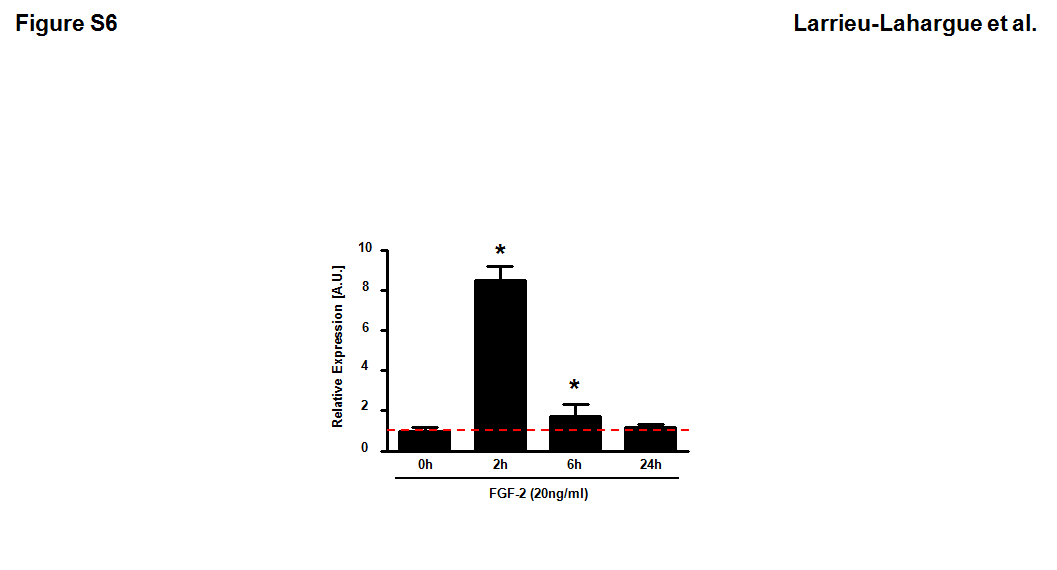

Supplement: Figure S6 — FGF-2 induces VEGF-C mRNA expression in 66c14 cancer cells. 66c14 cancer cells were incubated in the presence or the absence of recombinant FGF-2 (20 ng/ml) for different time durations, and total RNA retro-transcribed. VEGF-C mRNA expression was determined by quantitative PCR and expressed as fold change over control condition (red dashed line). (*p<0.05 versus control group, 0 h). (TIF) [file pone.0039540.s006.tif]

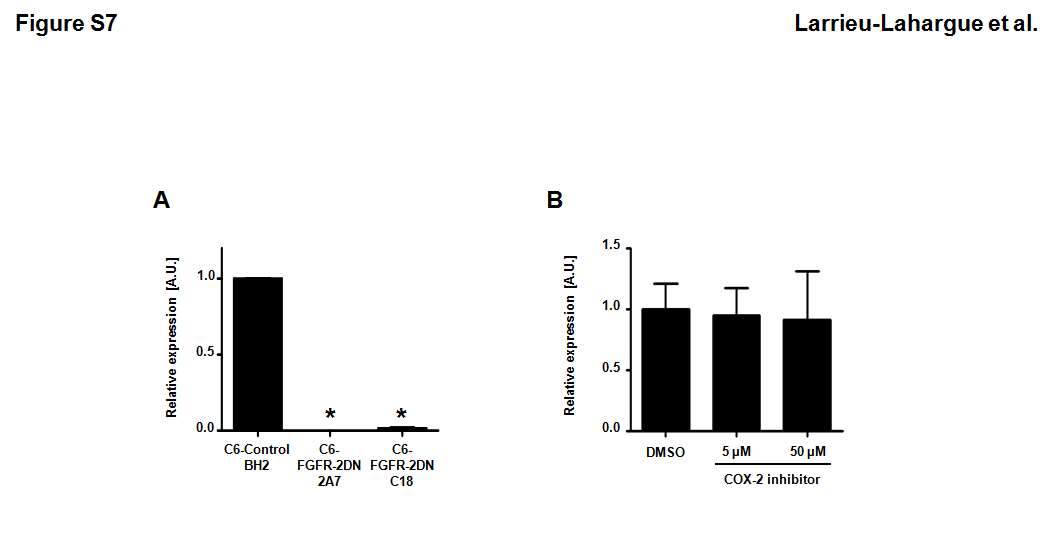

Supplement: Figure S7 — In C6 cancer cells, blockade of Fibroblast Growth Factor Signaling suppresses COX-2 independent VEGF-C expression. (A) VEGF-C mRNA expression is inhibited in rat C6 glioblastoma tumor cells expressing the FGFR-2DN (clones 2A7, C18) as compared to empty plasmid transfected control (control clone BH2). (B) VEGF-C mRNA expression is unchanged in C6 tumor cells treated with increasing doses of the COX-2 inhibitor NS-398. (*p<0.05 versus respective control group). (TIF) [file pone.0039540.s007.tif]

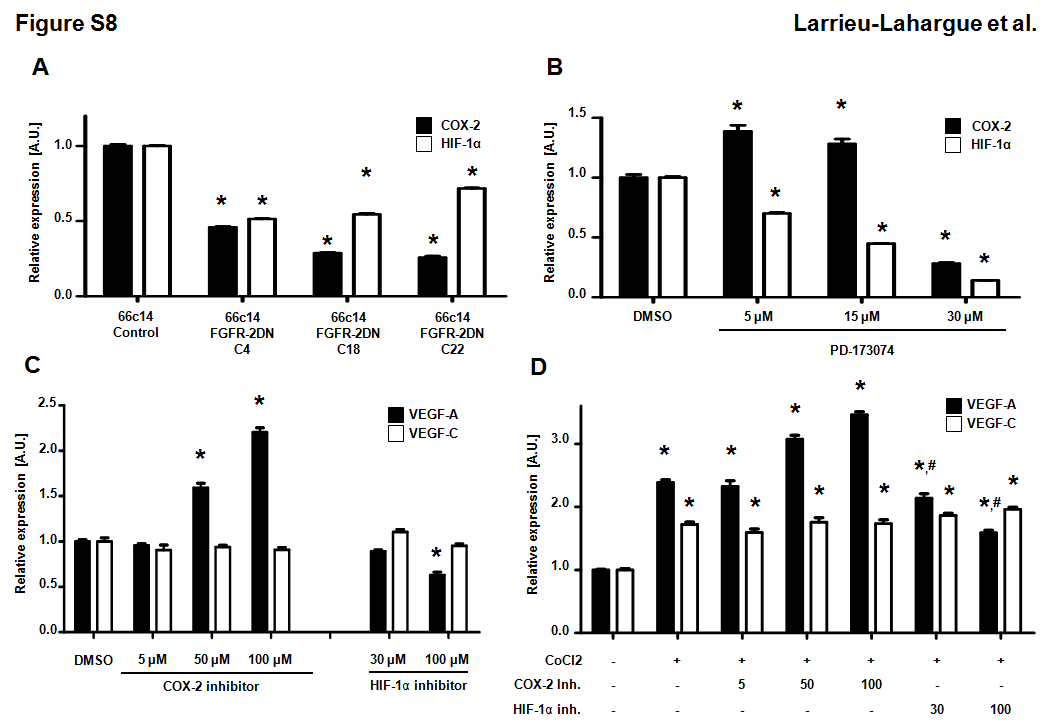

Supplement: Figure S8 — FGFR signaling stimulates VEGF-C expression independently of either COX-2 or HIF-1α. (A) COX-2 (black) and HIF-1α (white) mRNA expression is inhibited in FGFR-2DN-expressing 66c14 cells (clones C4, C18 and C22) compared to empty plasmid-transfected cells (control). (B) COX-2 (black) and HIF-1α (white) mRNA expression is inhibited in 66c14 cells treated with increasing doses of FGFR inhibitor PD-173074. (C) Left panel, VEGF-A (black) but not VEGF-C (white) mRNA expression is modified in 66c14 tumor cells treated with increasing doses of the COX-2 inhibitor NS-398. Right panel, the HIF-1α inhibitor 400083 decreases VEGF-A (black) but not VEGF-C (white) mRNA expression in 66c14 tumor cells, in normoxic conditions. (D) 66c14 cells were treated in the presence (+) or absence (-) of hypoxia inducer, cobalt chloride (10 mM), supplemented or not with various doses of cycloxygenase-2 (NS-398) or HIF-1α (400083) inhibitors (both concentrations in µM). VEGF-A (black) and VEGF-C (white) mRNA expression was then determined by qRT-PCR. VEGF-A, but not VEGF-C, hypoxia-induced mRNA expression was modified by both inhibitor treatments. (A–C: *p<0.05 versus respective control group; D: * and # p<0.05 versus cobalt chloride untreated and treated cells, without inhibitor, respectively). (TIF) [file pone.0039540.s008.tif]

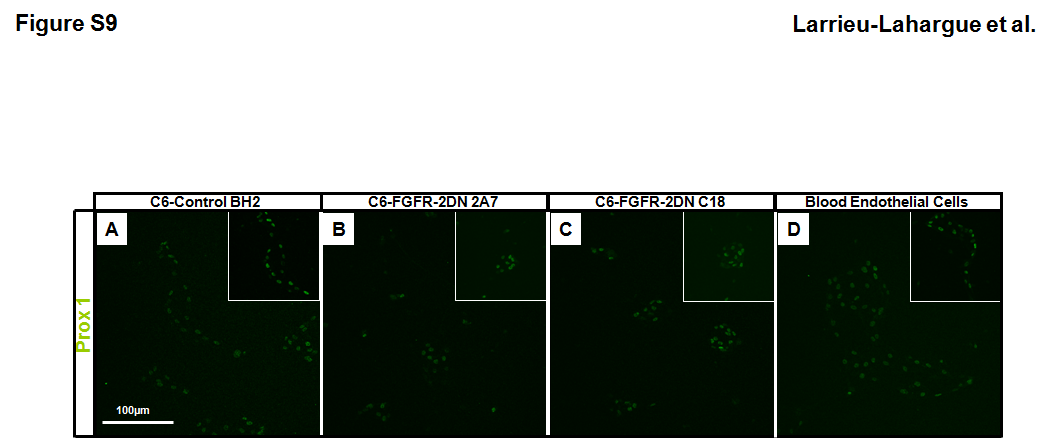

Supplement: Figure S9 — Expression of FGFR-2DN inhibits C6 cells-induced in vitro lymphangiogenesis. Prox-1-stained lymphatic-like vascular tubes are observed in the co-culture between lymphatic endothelial cells (HMVEC-dLys) and C6 control (BH2, A) or blood endothelial cells (HMVEC-d, D), while unorganized lymphatic endothelial cell clusters are detected in co-culture with FGFR-2DN-expressing C6 cells (2A7 and C18, B and C, respectively). (TIF) [file pone.0039540.s009.tif]

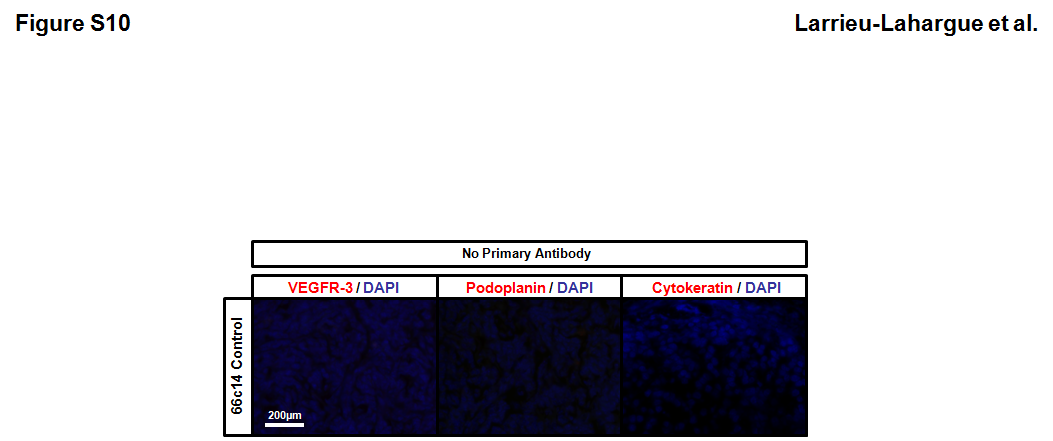

Supplement: Figure S10 — Immunohistochemical controls. Immunohistochemical labeling of 66c14 tumor controls observed in the absence of VEGFR-3 (left panel), Podoplanin (middle panel) or Cytokeratin (right panel) primary antibody. (TIF) [file pone.0039540.s010.tif]

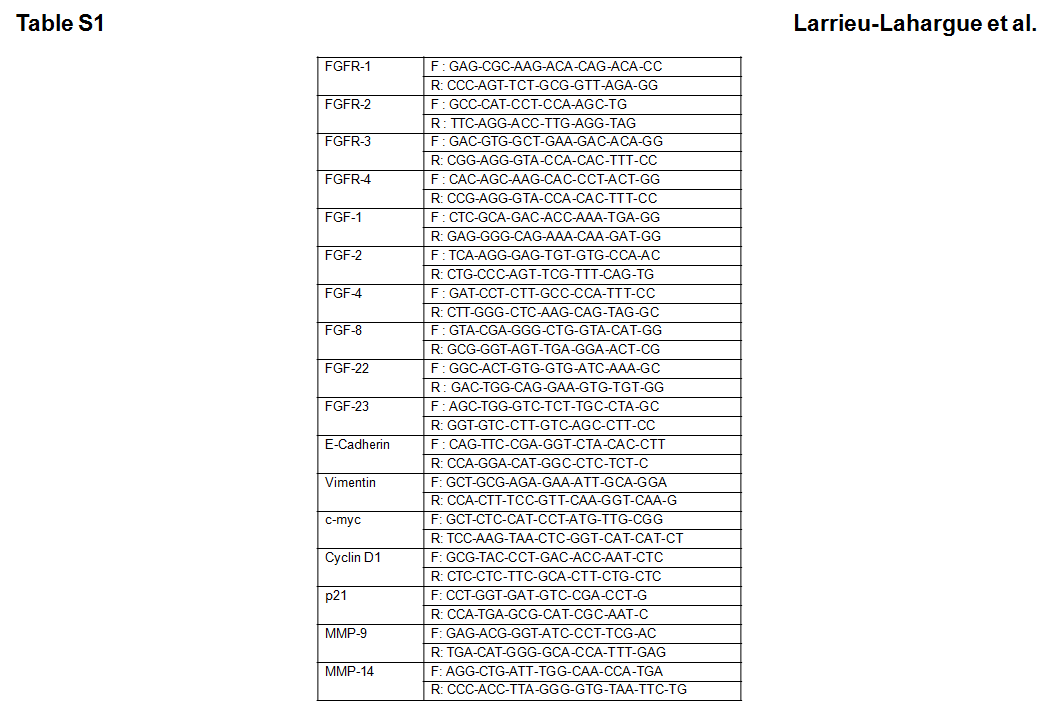

Supplement: Table S1 — Mouse primer sequences for standard and quantitative RT-PCRs. (TIF) [file pone.0039540.s011.tif]
